# Supplementary material for: Hematopoietic stem cells can differentiate into restricted myeloid progenitors before cell division in mice
Source: Nat Commun. 2018 May 15;9:1898. doi: 10.1038/s41467-018-04188-7 (PMC5954009; doi:10.1038/s41467-018-04188-7)
Supplement: Supplementary file 1 — Supplementary Information [file 41467_2018_4188_MOESM1_ESM.pdf]

Supplementary information

Hematopoietic stem cells can differentiate into restricted myeloid progenitors before cell division in mice

Grinenko et al.

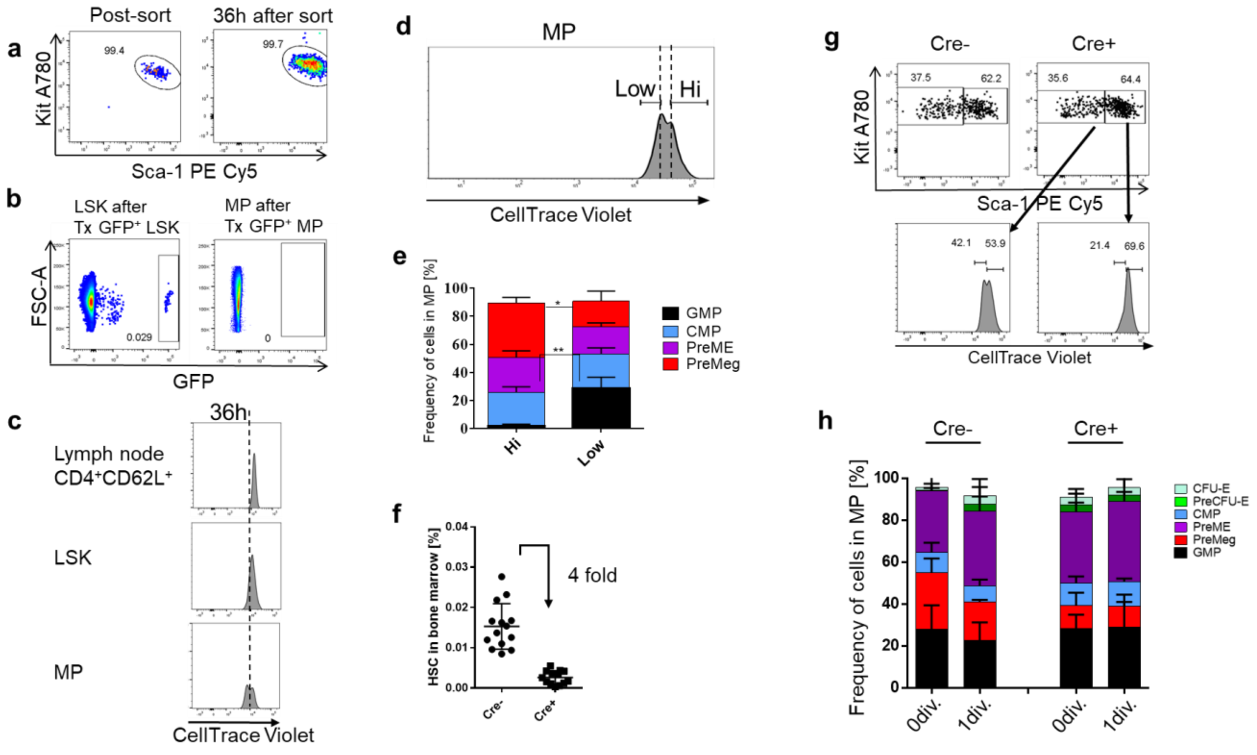

Supplementary Figure 1. Transplantation of HSCs into non-conditioned recipients and after depletion of endogenous HSCs. (**a**) HSCs (LSK CD48$^-$ CD41$^-$ CD150$^+$) were sorted and kept at +4^0 C in PBS/5% FCS solution for 36h and subsequently analyzed by FACS. (**b**) 3600 LSK and MPs were sorted from Ubc:GFP mice and transplanted into wild type mice. 36h after transplantation bone marrow was analyzed by FACS. (**c**) Analysis of the CellTrace Violet in undivided CD4$^+$CD62L$^+$ naive T cells, LSK, and MP cells at 36h after transplantation. CD4$^+$CD62L$^+$ naïve T cells (10^6), labeled with CellTrace Violet, were transplanted as controls for undivided cells. Lymph node donor cells were analyzed later, along with other donor cells. 13 independent experiments, 3-5 mice for each experiment. (**d**) Gating strategy for analysis of non-divided (CellTrace Viol Hi) and divided MPs (CellTrace Viol Low) cells. (**e**) Frequency of restricted MPs in CellTrace Violet Hi and divided CellTrace Violet Low cells. 13 independent experiments, 3-5 mice for each experiment. (**f**) Frequency of recipient HSCs (LSK CD48$^-$ CD150$^+$) 11-21 days after TAM induction and 36h after transplantation of labeled HSCs into HSC-

CreERT/R-DTA mice (Cre+ and Cre-). (**g**) Phenotype of donor derived cells, 36h after transplantation of CellTrace Violet labeled HSCs into HSC-CreERT/R-DTA. Representative plot with 5 mice per genotype, from 4 independent experiments (Cre- n=14, Cre+ n=13) (**h**) Frequency of restricted progenitors in undivided (0 div.) and divided (1 div.) donor MPs 36h after transplantation of HSCs into Cre+ and Cre- mice, pooled data from 4 independent experiments. (Cre- n=14, Cre+ n=13). Data are means +/- s.d.

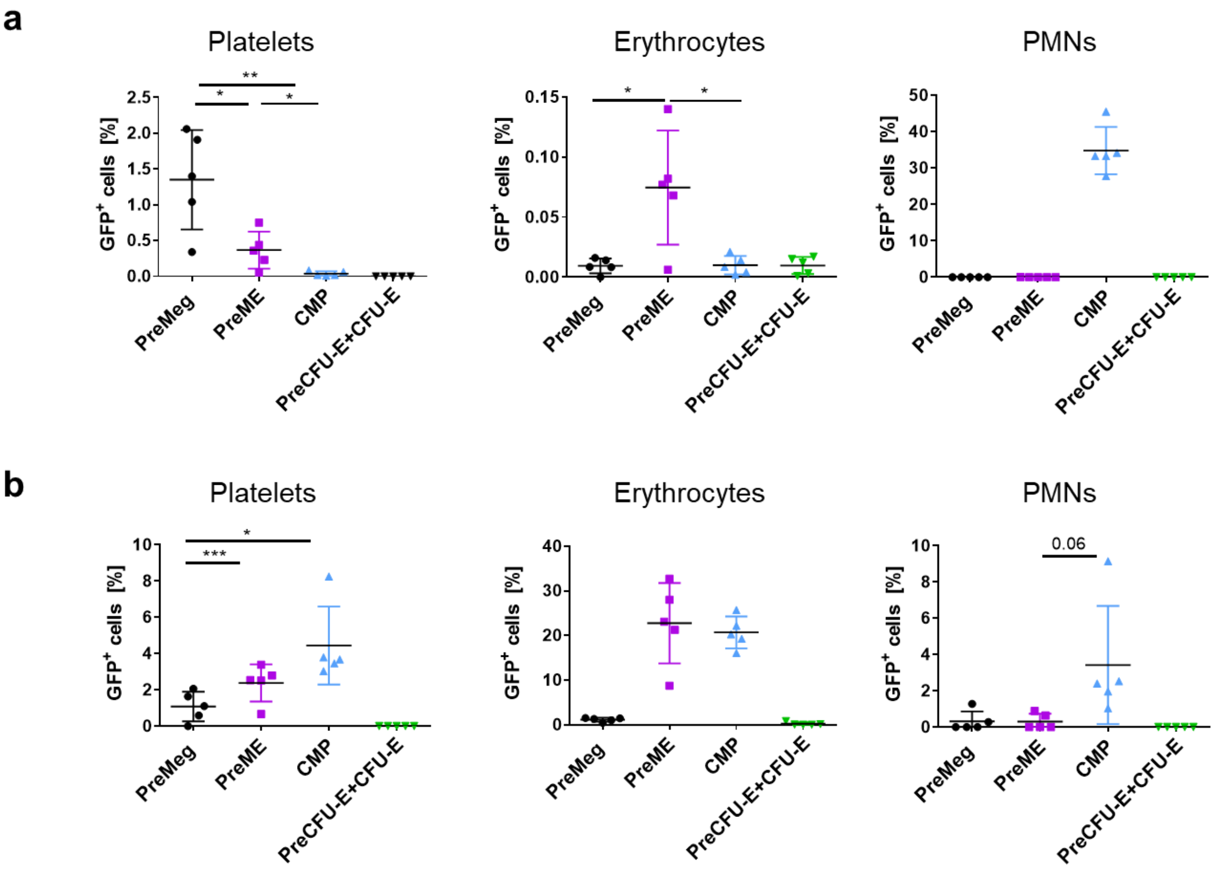

Supplementary Figure 2. Repopulation capacity of restricted MPs. Restricted myeloid progenitors (PreME: Lin$^-$ Sca1$^-$ Kit$^+$ CD16/32$^-$ CD150$^+$ CD41$^-$ CD105$^-$, PreMeg: Lin- Sca1$^-$ Kit$^+$ CD16/32$^-$ CD150$^+$ CD41$^+$ CD105$^-$, CMP: Lin$^-$ Sca1$^-$ Kit$^+$ CD16/32$^-$ CD150$^-$ CD41$^-$ CD105$^-$, PreCFU-E/CFU-E: Lin$^-$ Sca1$^-$ Kit$^+$ CD16/32$^-$ CD150$^{+/-}$ CD41$^-$ CD105$^+$). Cells from Ubc-GFP mice (10^4) were sorted and transplanted into lethally irradiated wild type mice together with $2x10^5$ total bone marrow competitor cells. **(a)** At 14d after transplantation and **(b)** at 26d after transplantation, donor cell contribution to PMNs (CD11b$^+$ Gr1$^+$), erythrocytes (Ter119$^+$) and platelets (Ter119$^-$ CD41$^+$) were analyzed. n=5, 2 independent experiments. Statistical significance was determined using unpaired Student's t-test *($p<0.05$), **($p<0.005$), ***($p<0.0001$). Data are means +/- s.d.

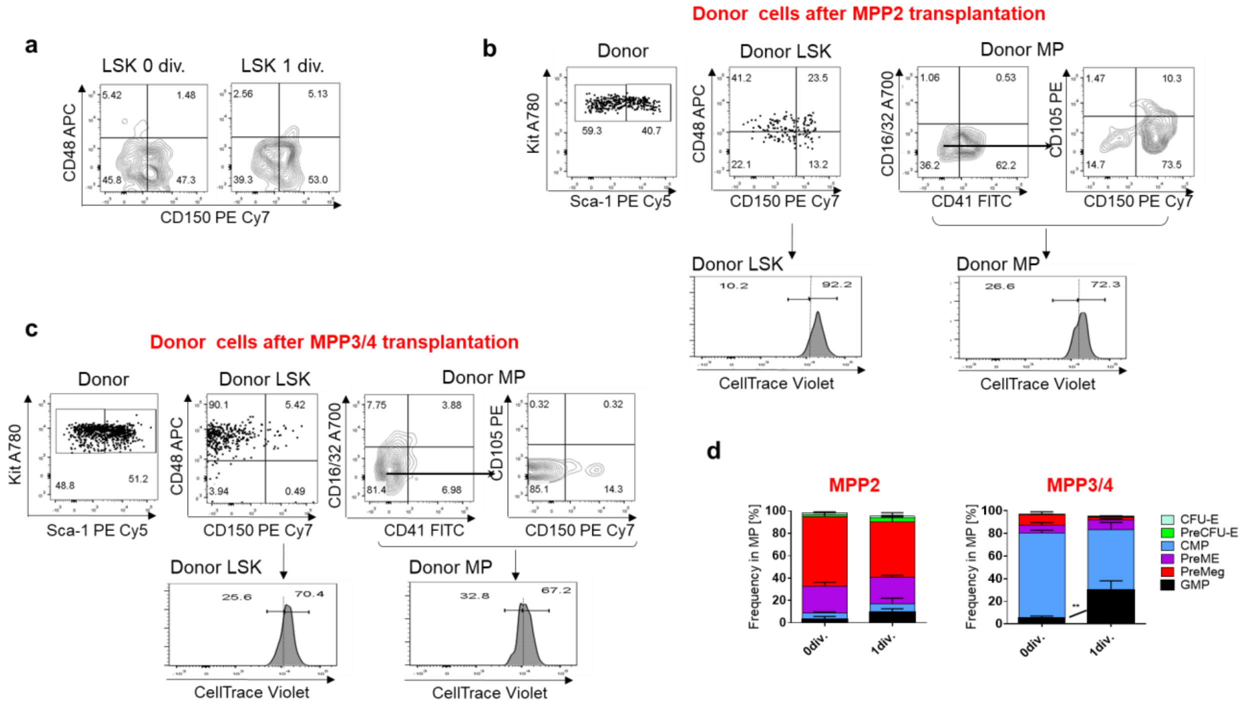

Supplementary Figure 3. Analysis of HSC and MPP progeny after transplantation into unconditioned recipients. **(a)** Phenotype of undivided and divided donor LSK cells at 36h after transplantation. 5000 MPP2 (LSK CD48^{+} CD150^{+}) **(b)** or 10,000 MPP3/4 cells (LSK CD48^{+} CD150^{-}) **(c)** were labeled with CellTrace Violet and transplanted into non-irradiated recipients. Donor cells were analyzed 36h after transplantation. **(d)** Quantification of restricted progenitor's frequency in donor's undivided (0 div.) and divided (1 div.) MP population. Pooled data from 3 independent experiments, n=3 for each. Statistical significance was determined using unpaired Student's t-test **(p=0.009). Data are means +/- s.d.

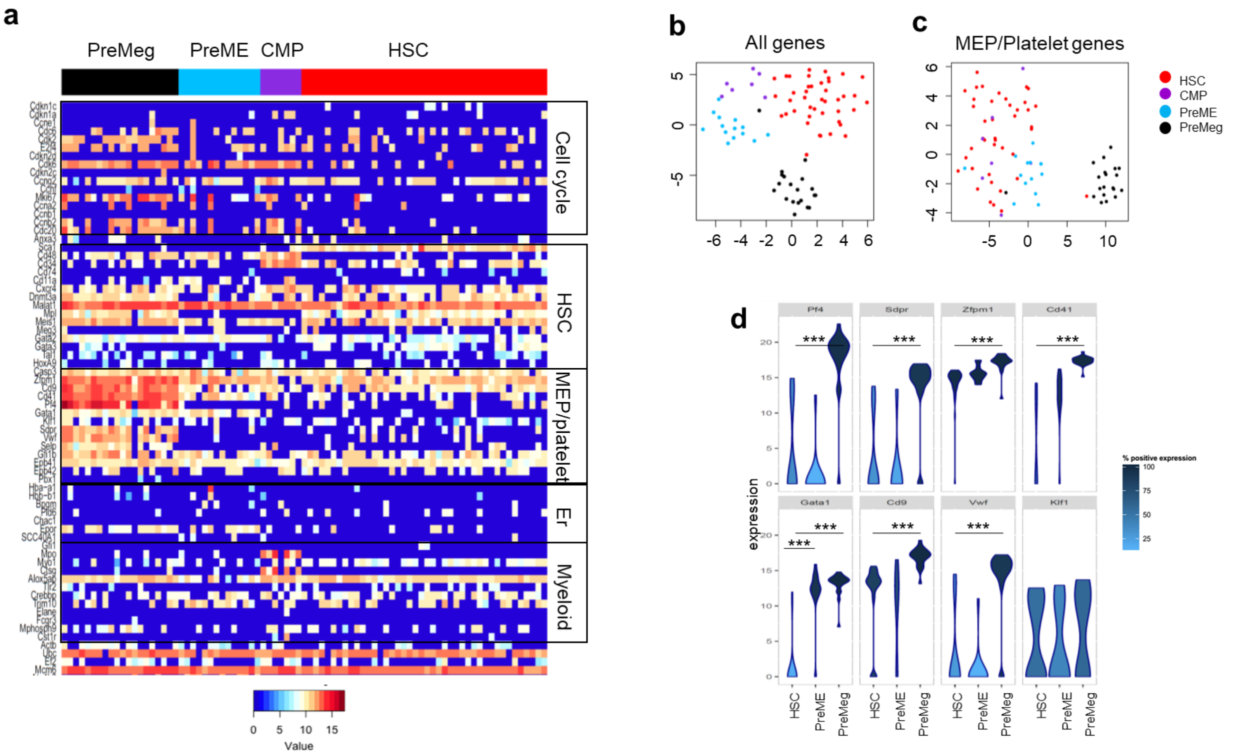

Supplementary Figure 4. Single-cell expression analysis before transplantation. Single cells: 42 HSCs (LSK CD41$^-$ CD48$^-$ CD150$^+$), 15 PreME: Lin$^-$ Sca1$^-$Kit$^+$ CD16/32$^-$ CD150$^+$ CD41$^-$ CD105$^-$, (one single cell was excluded as it didn't express any of the tested genes) 20 PreMeg: Lin$^-$ Sca1$^-$ Kit$^+$ CD16/32$^-$ CD150$^+$ CD41$^+$ CD105$^-$, and 7 CMP: Lin$^-$ Sca1$^-$ Kit$^+$ CD16/32$^-$ CD150$^-$ CD41$^-$ CD105$^-$, were sorted and single-cell qPCR performed. **(a)** Heat map representing gene expression analyses. Each row corresponds to a specific gene and each column corresponds to a particular donor single cell. Heat map colors represent expression levels of individual genes (dCt). **(b)** t-SNE plot for all analyzed genes and cells, axes display arbitrary units. **(c)** t-SNE plot for MEP/Platelet genes for all cells, axes have arbitrary units. **(d)** Violin density plots for the most differently expressed MEP/Platelet genes. Y-axis represents gene expression. The horizontal width of the plot shows the density of the data along the Y-axis. Statistical significance was determined using the Hurdle model. *($p<0.05$), **($p<0.01$), ***($p<0.0001$), exact p value in Supplemental Tables S4-5. Cells were sorted from 3 mice.

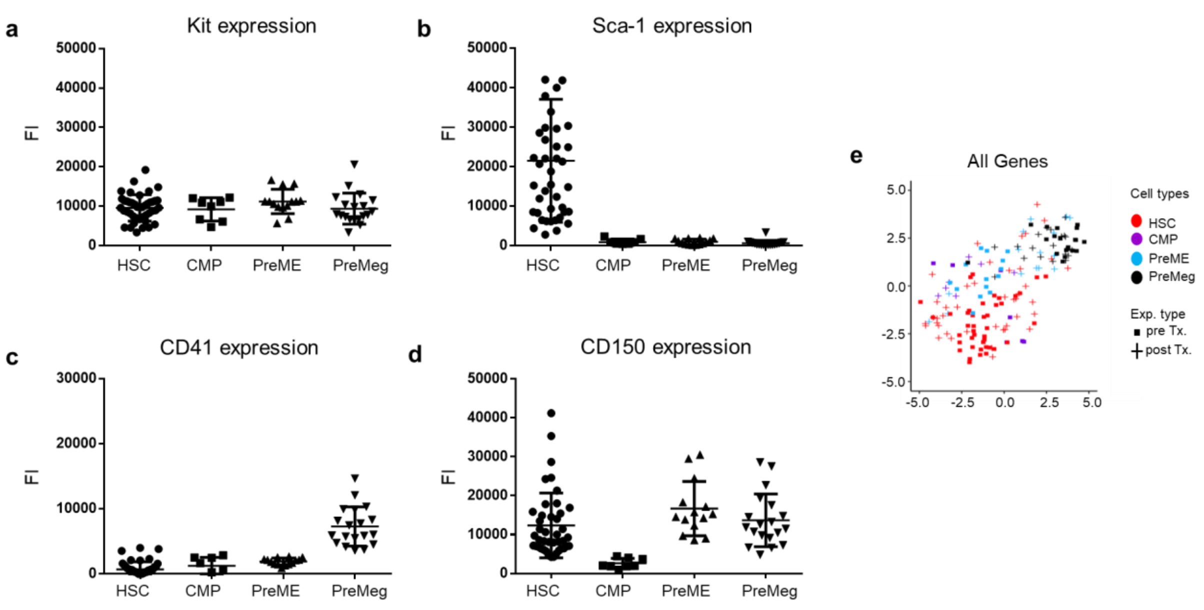

Supplementary Figure 5. Separation of cells based on fluorescent intensity (FI) from index sort data. **(a)** Kit expression. **(b)** Sca-1 expression. **(c)** CD41 expression. **(d)** CD150 expression. Each dot represents a single cell. Data are means +/- s.d. **(e)** t-SNE plot for all analysed genes for all cells before transplantation and undivided donor cells at 36h after transplantation. Axes display arbitrary units.

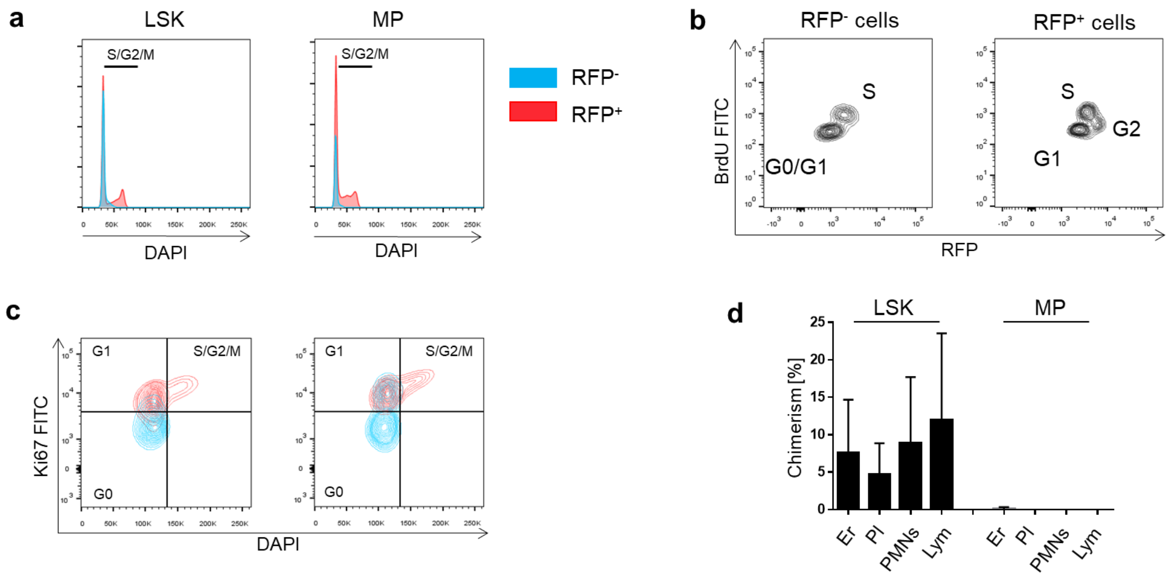

Supplementary Figure 6. Verification of RFP expression in the hematopoietic compartment of $Ki67^{RFP}$ knock-in mice based on cell cycle progression. RFP^+ and RFP^- Lin^- Kit^+ cells were sorted from $Ki67^{RFP}$ knock-in mice and **(a)** DNA content (DAPI) of RFP^- (blue) and RFP^+ (red) LSK and MP cells was analyzed. **(b)** Sorted RFP^+ and RFP^- Lin^- Kit^+ cells were incubated in culture with 10µM BrdU for 3.5h, cells were fixed, and BrdU incorporation analyzed and compared with RFP expression. **(c)** Sorted RFP^+ and RFP^- Lin^- Kit^+ cells were stained with an anti-KI67 antibody. RFP^- cells represent cells in G0/G1 phase of the cell cycle, while RFP^+ represents G1/S/G2/M cells. 2 independent experiments, n=2 for each. **(d)** Quantification of peripheral blood analysis 16 weeks after transplantation from 2 independent experiments, n=4. Statistical significance was determined using an unpaired Student's t-test *($p<0.05$). Data are means +/- s.d.

	name		primer	gene type		name		primer	gene type
1	*Ubc*	F	CCGCCTTCTTCAGTATGCAGA	house keeping gene	36	*HoxA9*	F	CCGGACGGCAGTTGATAGAG	HSC
		R	TGCTTTCGGATATAGCCCTGG				R	CTTCTTCCGAGTGGAGCGAG	
2	*Ef2*	F	CTTTGAACGGAGGGCAGATG	house keeping gene	37	*Casp3*	F	TGGTGATGAAGGGGTCATTTATG	MEP/PreMeg
		R	CGCCTCTGTTTCCGCTTTG				R	TTCGGCTTTCCAGTCAGACTC	
3	*Actb*	F	GGCTGTATTCCCCTCCATCG	house keeping gene	38	*Zfpm1*	F	ACCAGAGCCTTATCCCCTGA	MEP/PreMeg
		R	CCAGTTGGTAACAATGCCATGT				R	AGGGCGTCATCCTTCCTGTA	
4	*Mcm 6*	F	GGACGAGTCGGCACTAAAGA	house keeping gene	39	*Cd9*	F	GGGATTGTTCTTCGGGTTCCT	MEP/PreMeg
		R	AACGTGATCGTAGTGTGTGAGA				R	GGGTTCATCCTTGCTCCGTA	
5	*Cdkn1c (p57)*	F	CAGCCAGCCTTCGACCAT	cell cycle	40	*Itga2b (CD41)*	F	GAGTTTTCGCGGAGACAAGC	MEP/PreMeg
		R	TACGCGCTATCACTGGGAAG				R	TTCTCAATTGGAACGCGAGC	
6	*Cdkn1a (p21)*	F	CGG TGT CAG AGT CTA GGG GA	cell cycle	41	*Pf4*	F	TCCCGAAGAAAGCGATGGAG	MEP/PreMeg
		R	ATC ACC AGG ATT GGA CAT GG				R	CCATTCTTCAGGGTGGCTATGA	
7	*Ccne1 (Cyclin E1)*	F	GGCAAATGTGGCCGTGTTTT	cell cycle	42	*Gata1*	F	TATGGCAAGACGGCACTCTAC	MEP/PreMeg
		R	GGATGAAAGAGCAGGGGTCC				R	GGTGTCCAAGAACGTGTTGTT	
8	*Cdc6*	F	TTTCGGAAGTTGATGGGAAC	cell cycle	43	*Klf1*	F	CTGCGCCAAAGCCTATTACA	MEP/PreMeg
		R	GGGTCAAAAGCAGCAAAGAG				R	GGCGAACTTCTTGTCACAGC	
9	*Cdk2*	F	AAGAAGATCCGGCTCGACAC	cell cycle	44	*Sdpr*	F	CCAGGTGAAGAGACTGGAGAAC	MEP/PreMeg
		R	ACATCCAGCAGCTTGACGATA				R	TCCTTCACAAACACACTGGCA	
10	*E2f4*	F	CCCTTCTACCTCCTTTGAGCC	cell cycle	45	*Vwf*	F	TGTATGGGCAAGATGAACCCTT	MEP/PreMeg
		R	CATGCACTCTCTCGTGGGG				R	GAAGTCGTCCCCAAAGAGGC	
11	*Cdkn2d (p19)*	F	GCCTTGCAGGTCATGATGTTT	cell cycle	46	*Selp*	F	CCCCATTCTGTGAAGCGTTG	MEP/PreMeg
		R	GAACCTTCAGGGTGTCCAGG				R	ACCAAGAGCGAGCCTTCATC	
12	*Cdk6*	F	CCTTACCTCGGTGGTCGTC	cell cycle	47	*Gfi1b*	F	GTTGCTGAACCAGAGCCTTC	MEP/PreMeg
		R	GAACTTCCACGAAAAAGAGGCT				R	CAGGAGAAGCTGGGCTTGTA	
13	*Cdkn2c (p18)*	F	GGGGACCTAGAGCAACTTACT	cell cycle	48	*Epb4.1*	F	GCAGGTTCGAGGTGTTCCTT	MEP/PreMeg
		R	AAATTGGGATTAGCACCTCTGAG				R	AATGTCCTGCCGAAGCTGAA	
14	*Ccng2 (Cyclin G2)*	F	TTCCTGAGCTGCCCACTATC	cell cycle	49	*Epb4.2*	F	ACTGCATTCAGGAAGAGCCC	MEP/PreMeg
		R	AAGGTGCACTCCTGATCGC				R	CAACAAGGCGCCCACAAC	
15	*Ccnf (Cyclin F)*	F	CGCGCTTGAGAGCGGT	cell cycle	50	*Pbx1*	F	ACAGTCTCCCAGGTGGATACC	MEP/PreMeg
		R	GTAGGATAACAGAAACACTTGGCAC				R	CCTGCCAACCTCCATTAGCA	
16	*Mki67 (Ki 67)*	F	ATCATTGACCGCTCCTTTAGGT	cell cycle	51	*Hba-a1*	F	CAACTTCAAGCTCCTGAGCCA	Erythroid
		R	GCTCGCCTTGATGGTTCCT				R	AACGGTACTTGGAGGTCAGC	
17	*Ccna2 (Cyclin A2)*	F	AAGAAACAGAGTGTGAAGATGCC	cell cycle	52	*Hbb-b1*	F	TGCTTCTGACATAGTTGTGTTGAC	Erythroid
		R	AGACATATCCATAGCATGTGGTGA				R	AACCAGCAGCCTGCCC	
18	*Ccnb1 (Cyclin B1)*	F	GTGAGTGACGTAGACGCAGA	cell cycle	53	*Bpgm*	F	GCTGTTCCTCGGCTAGGTAC	Erythroid
		R	CACTTCACGACCCTGTAGGT				R	AGCCTAAAGAAACGGCAGCT	
19	*Ccnb2 (Cyclin B2)*	F	CGACGGTGTCCAGTGATTTG	cell cycle	54	*Pld6*	F	CGCAAGGCAGGGATACAGG	Erythroid
		R	TCTGAGGTTTCTTCGCCACC				R	GTGTGGTCCAGTTGAGGGAG	
20	*Cdc20*	F	GTGCTCCATCCTCTGGTCTC	cell cycle	55	*Chac1*	F	GGAAGCCGGACTTTGCCTAT	Erythroid
		R	ATTGTAAGACCCAGGACCCG				R	CGGTCTTCAAGGAGGGTCAC	
21	*Anxa3*	F	CAGGTCAAGCAGGCAGATGA	cell cycle, apoptosis	56	*Atp1b2*	F	CACACCAGGCTTGATGATTCG	Erythroid
		R	GCAGAGCTTTCCGGAAGTCT				R	CTGTGCTTGGATGGAGTCGT	
22	*Ly6A (Sca1)*	F	CCTTCTCTGAGGATGGACACTT	HSC	57	*Epor*	F	GGGCTCCGAAGAACT	Erythroid
		R	AGTCCCTGAGCTCTTTCTGC				R	ATGACTTTCGTGACT	
23	*Cd48*	F	TGCCAAGTCAGCAATCCTGT	HSC	58	*SCC40A1*	F	GTCTATGGACTGGTGGTGGC	Erythroid
		R	ACTAGCCAAGTTGCAGTCCA				R	GTGACGTCTGGGCCACTTTA	
24	*Cd34*	F	ACATCACCCACCGAGCCATA	HSC	59	*Gfi1*	F	TCCGAGTTCGAGGACTTTTG	Myeloid
		R	AAACTCCTCACAACTAGATGCTTCA				R	CATGCATAGGGCTTGAAAGG	
25	*Cd74*	F	CACCGGTGTCTCTGTCCTG	HSC	60	*Mpo*	F	ACTGCAACAACAGACGAAGC	Myeloid
		R	GGAGGTGATGGTCAGCTTGT				R	GAAGCCATTGCGATTGACTCC	
26	*Cd11a (ItgaL)*	F	ACTTCCACTTCCCGATCTGC	HSC	61	*Myo1F*	F	AACAATTCCAGCCGCTTTGG	Myeloid
		R	CTCAGGATAGGCTGCATGGC				R	GCATGACCACACGAGACTTTTC	
27	*Cxcr 4*	F	ATGGAACCGATCAGTGTGAGT	HSC	62	*Ctsg*	F	GGGTTTCTGGTGCGAGAAGA	Myeloid
		R	TGAAGTAGATGGTGGGCAGG				R	AGTTGCTGGGTCCTTTCTCG	
28	*Dnmt3a*	F	AAACTTCGGGGCTTCTCCTG	HSC	63	*Alox5ap*	F	CGTTCTTTGCCCACAAGGTG	Myeloid
		R	GCTTCTCCACACACACCACT				R	GGGTACGCATCTACGCAGTT	
29	*Malat 1*	F	CCTTTGCAGAGCCTCATTTC	HSC	64	*Tlr2*	F	GGGGCTTCACTTCTCTGCTT	Myeloid
		R	CCTCCACATTCCAGGAAAAA				R	AGAGCCAAAGAGCTCGTAGC	
30	*Mpl*	F	CTGTATGCCTACCGAGGAGAG	HSC	65	*Crebbp*	F	AAGATGCGCAACCTGGAGAC	Myeloid
		R	TCTGGTTGAGGGACACATTCTT				R	GCTTGAACAAGTTCCGCAGG	
31	*Meis 1*	F	TAGACCAGTCCAACCGAGCA	HSC	66	*Trim 10*	F	TGAAACCAGAAAGTGCCGGA	Myeloid
		R	CATTCCACTCATAGGTCCTGGC				R	GCTGGCTCATAGTCCAACTCA	
32	*Meg3*	F	ACCATCCCTTGCTGGTTCTT	HSC	67	*Elane*	F	CGTCGTGTGAACGTATGCAC	Myeloid
		R	GGAAGGACGATGGCTAGGA				R	ATCCAGATCCACAGCCTCCT	
33	*Gata3*	F	CTTATCAAGCCCAAGCGAAG	HSC	68	*Fcgr3 (CD16/32)*	F	GCTGCTGTTTGCTTTTGCAG	Myeloid
		R	CCCATTAGCGTTCCTCCTC				R	TCCCTTCGCACATCAGTGTC	
34	*Gata2*	F	CCTGGTTCCCAAGACACAGT	HSC	69	*Mphosph (MPP9)*	F	AGACCAGATTGAGGCAGCAC	Myeloid
		R	CTGTGCTGGCTCCATGTAGTT				R	GAGCCCAGTTCACGGTTGAT	
35	*Tal1 (Scl)*	F	GTTGTTTACCGCGCAGGAC	HSC	70	*Csf1r*	F	CAGTACTTCAGGGCCGTCTC	Myeloid
		R	AGAGACCTACTCGGCTGGTT				R	TCGGCTTCTGCTTGTACTTGT	

Supplementary Table 1. Primer panel for single-cell qPCR

Gene	Test	p value
Sca1	hurdle	2.65E-05
Pf4	hurdle	0.010728
Gata1	hurdle	0.017086
Zfpm1	hurdle	0.061063

Supplementary Table 2. Analysis of gene expression in HSCs and PreMEs after transplantation

Gene	Test	p value
Pf4	hurdle	3.87E-08
Itga2b.Cd41	hurdle	2.92E-07
Cd9	hurdle	5.89E-07
Gata1	hurdle	7.81E-06
Zfpm1	hurdle	1.75E-05
Sdpr	hurdle	2.46E-05
Cdk6	hurdle	2.66E-05
Ly6A.Sca.1	hurdle	7.24E-05
Vwf	hurdle	0.000764
E2f4	hurdle	0.001107
Actb	hurdle	0.003485
Mki67	hurdle	0.005445
Cdkn2d	hurdle	0.009814
Klf1	hurdle	0.009814

Supplementary Table 3. Analysis of gene expression in HSCs and PreMegs after transplantation

Gene	test	value
Sca1	hurdle	1.05E-12
Gata1	hurdle	1.97E-07
Mpl	hurdle	1.76E-06
Atp1b2	hurdle	0.000225
Cd48	hurdle	0.000865
Cdk6	hurdle	0.001021
Alox5ap	hurdle	0.005581
Cd11a	hurdle	0.008792
Gata3	hurdle	0.008966
Dnmt3a	hurdle	0.02042
Gata2	hurdle	0.062408
Cd41	hurdle	0.07717
Epor	hurdle	0.07717

Supplementary Table 4. Analysis of gene expression in HSCs and PreMEs before transplantation

Gene	test	value
Cd41	hurdle	7.19E-19
Sca1	hurdle	3.12E-13
Gata1	hurdle	9.71E-13
Pf4	hurdle	1.27E-12
Cd9	hurdle	1.28E-12
Sdpr	hurdle	1.98E-10
Zfpm1	hurdle	7.41E-10
Cdk6	hurdle	1.34E-09
Vwf	hurdle	3.79E-09
Mki67	hurdle	6.69E-07
Cd48	hurdle	8.07E-06
Selp	hurdle	9.42E-06
Cdc20	hurdle	5.64E-05
Epor	hurdle	7.92E-05
Ccnb2	hurdle	0.000178
Cdk2	hurdle	0.003142

Supplementary Table 5. Analysis of gene expression in HSCs and PreMegs before transplantation
